# A combined exercise model for improving muscle strength, balance, walking distance, and motor agility in multiple sclerosis patients: A randomized clinical trial

**Published:** 2016-07-06

**Authors:** Bahram Sangelaji, Mohammadreza Kordi, Farzaneh Banihashemi, Seyed Massood Nabavi, Sara Khodadadeh, Maryam Dastoorpoor

**Affiliations:** 1 Department of Physiotherapy, School of Physiotherapy, Otago University, Dunedin, New Zealand AND Iranian Multiple Sclerosis Society Rehabilitation Centre, Tehran, Iran; 2 Department of Exercise Physiology, School of Physical ‎Education and Sport Sciences, University of Tehran, Tehran, Iran; 3 Mother and Child Welfare Research Center, Hormozgan University of Medical Sciences, Bandar Abbas, Iran; 4 Department of Neurology, School of Medicine, Mostafa Khomeini Hospital, Shahed University of Medical Sciences, Tehran, Iran; 5 Modeling in Health Research Center, Institute for Futures Studies in Health, Kerman University of Medical Sciences, Kerman, Iran

**Keywords:** Multiple Sclerosis, Exercise Therapy, Aerobic Exercise, Resistance Training

## Abstract

**Background:** Multiple sclerosis (MS) is a neurological disease with a variety of signs and symptoms. Exercise therapy has been shown to improve physical functions in MS. However, questions about an optimal exercise therapy remain. In this regard, we suggest a combined exercise therapy including aerobic and resistance exercises for MS patients. The study is designed to observe, test and compare the effects of proposed combined exercises on strength, balance, agility, fatigue, speed, and walking distance in people with mild to moderate MS [0 < expanded disability status scale (EDSS) < 5].

**Methods:** A total of 40 people with relapse-remitting MS (16 male, 0 < EDSS < 5) were randomized into one of the four groups (3 intervention and one control). The intervention consisted of various combinations of aerobic and resistance exercises with different repetition rates. Pre- and post-intervention scores of fatigue severity scale (FSS), timed up and go (TUG) test, 6-minute walk test (6MWT), 10- and 20-MWT, Berg balance scale (BBS), and one repetition maximum (1RM) test were recorded and analyzed.

**Results:** For most tests, post-intervention values of the group 1, with 3-aerobic and 1-resistance exercises, were significantly higher compared to control group (P < 0.050). However, no significant progression was observed in the other two intervention groups.

**Conclusion:** A combination of three aerobic exercises with one resistance exercise may result in improved balance, locomotion, and endurance in MS patients.

## Introduction

Multiple sclerosis (MS) is an inflammatory demyelinating disorder of the central nervous system with both inflammation and neurodegeneration outcomes such as inflammatory attacks.^1^ Adults between 18 and 40 are commonly affected by MS with a relapsing-remitting and sometimes a steady progression course.^2^^,^^3^ The most common symptoms of MS include weakness, fatigue, and imbalance.^2^^,^^3^ Balance impairment, which can lead to falls and injuries, is reported in 78% of people with MS.^4^ There is no uniform and/or well-established pharmacologic method to resolve imbalance, fatigue and weakness in MS. However, rehabilitation methods may be helpful.^5^^,^^6^ A number of studies have reported the benefits of exercise and physical activity.^7^ On one hand, power exercises can ameliorate muscle weakness and improve coordination which, in turn, can improve balance, agility and decrease muscle spasticity.^7^^-^^10^ Further, studies have reported increased muscle strength and functional capacity, using different power exercises in people with MS.^11^^-^^13^ On the other hand, aerobic exercise has been shown to significantly decrease fatigue^14^ and increase walking distance^15^ or speed.^16^

Although several studies have approved the efficacy of exercise to improve balance in people with MS, each has followed a different exercise protocol and yielded different results. A gradual progression from simple exercises such as stationary biking or weight lifting, to a combination of exercises has been reported to be beneficial.^6^ For instance, combined exercises improved patients’ balance^17^^-^^19^ as well as endurance.^20^^-^^22^

Although combined exercises have proved effective,^19^^,^^23^ their complexity may force patients and professionals to do them in well-equipped centers. Besides, these types of interventions were conducted on patients with mild relapsing-remitting MS, with an expanded disability status scale (EDSS) of < 3.5. To best of our knowledge, there are no studies on the effects of exercise in MS patients with moderate to severe disability and in progressive type.^5^^,^^8^ Hence, the objective of this study is to observe, test and compare the effects of proposed combined exercises on strength, walking speed, walking distance, balance, agility and fatigue, in mild to moderate people with MS (0 < EDSS < 5).

## Materials and Methods

This is a case-control randomized clinical trial. Due to obvious limitations, only those assessing the outcomes were blinded to group assignment. Members of Iranian MS Society (IMSS) were referred to IMSS physiotherapy center in Tehran, Iran, by their neurologists for rehabilitation program from September until November 2012. Demographic information of all patients was recorded in the center database, and those met the inclusion/exclusion criteria were advised to participant in the study. Finally, 40 people with MS were recruited and randomly assigned to four groups: three experimental and one control group. To avoid confounding effects, the four groups were matched on group characteristics [namely age, gender, body mass index (BMI), and social status].

***Inclusion/exclusion criteria***

The inclusion criteria include:

1. Definite relapse-remaining MS (RRMS)
2. Adults between 18 and 50 years of age
3. An EDSS level of 0-5
4. Right-handed
5. No history of systemic disease, concomitant neurological disorders, epilepsy, heart diseases, anemia, or severe depression.

The exclusion criteria include:

1. Under treatment with corticosteroid (in relapse time), or a history of recent attack (< 3 months)
2. Participants who completed < 30 sessions of exercise for any reason.

Participants were randomly assigned to four groups:

1. Group 1, which performed 1 aerobic exercise training and 3 resistance exercise training sessions per week
2. Group 2, which performed 2 aerobic exercise training and 2 resistance exercise training sessions per week
3. Group 3, Group 1, which performed 3 aerobic exercise training and 1 resistance exercise training session per week
4. Control group: All participants voluntarily filled the informed consent. Baseline scores were recorded within 5 days before the intervention and post-test scores were recorded exactly 72 hours after the end of the protocol for each group.

Outcome measures evaluated in this study are defined and measured as below:

1. One repetition maximum (1RM) test: To measure strength (heaviest weight a person can lift using quadriceps and hamstring muscles at first attempt)^24^^,^^25^
2. Berg balance scale (BBS): To measure balance^26^
3. Timed up and go (TUG) test: To measure agility^27^^,^^28^
4. 10-minute walk test (10MWT) and 20MWT: To measure speed of movement^29^
5. 6MWT: To measure the endurance and functional capacity^30^^,^^31^
6. Fatigue severity scale (FSS): To measure fatigue^30^^,^^31^
7. BMI: Weight in kilograms divided by the square of height in centimeters.^32^

A JEXERS^®^ exercise machine with a tolerance of 1 kg was used to measure the quadriceps and hamstring strength based on 1RM. Furthermore, a metal meter was used to measure the height of subjects in centimeters and a G200 BEURER^®^ (China) digital scale with 100 g tolerance to measure the weight of cases. 

In addition, BBS test was based on the Farsi version, which is a standard device in the IMSS rehabilitation center.^26^ To test for the walking speed, a running track in the gymnasium of rehabilitation center was measured and marked exactly at 10 and 20 m.^33^ For the TUG test, as mentioned in the manual, a chair, and a digital chronometer were used. The 6MWT was performed in a big gymnasium out of rehabilitation center.

Participants in the intervention groups performed exercises in groups. However, due to space and time limitations, it was not possible for all the groups to do the exercises simultaneously.^34^ Each group had four exercise sessions per week for 8 weeks (32 sessions). The interventions consisted of three stages per session: Stage 1: Warm up, Stage 2: main intervention and Stage 3: cool down.

- Stage 1: in this stage, one of the trainers demonstrated simple stretches for the neck, upper/lower extremities, and the trunk. Subjects were asked to follow.
- Stage 2: during the main interventional stage, each group followed their own program. For example, group 1 patients practiced individually tailored resistance exercises one session each week. For the next three sessions of the week, participants did two aerobic exercises: stationary bike and treadmill. Table 1 illustrates the workout routine for both resistance and aerobic exercises. For groups 2 and 3, the exercise sessions changed to 2 resistance/2 aerobic and 3 resistance/1 aerobic sessions per week, respectively. Maximum heart rate (MHR) of each person was tracked to prevent exhaustion while biking or using treadmill. During the 1^st^ week, the aerobic exercise begun with about 40% of MHR and 10 minutes per each device, then it gradually increased up to 70% of MHR and 20 minutes for each aerobic exercise. In addition, strength exercises started with 50% of 1RM with 10 repetitions of 3 sets and increased up to 70% of 1RM with 10 repetitions and three sets of exercise for each flexor or extensor of both knees.^24^^,^^25^
- Between two aerobic activities, bike and treadmill, and resistance sessions, extensors and flexors of the both knees, patients had a 10-minute and 5-minute inactive rest, respectively. If a patients’ heart rate reached above the limit, the exercise was stopped and the participant had to rest until the heart rate decreased (Tables 1 and 2).

**Table 1:** Endurance exercises mode

**Exercise types**	**Week 1**	**Week 2**	**Week 3**	**Week 4**	**Week 5**	**Week 6**	**Week 7**	**Week 8**
Cycling	10 minutes	10 minutes	15 minutes	15 minutes	15 minutes	20 minutes	20 minutes	20 minutes
	40%	50%	50%	55%	55%	55%	60%	70%
	MHR	MHR	MHR	MHR	MHR	MHR	MHR	MHR
Rest	10 minutes	10 minutes	10 minutes	10 minutes	10 minutes	10 minutes	10 minutes	10 minutes
	Inactive	Inactive	Inactive	Inactive	Inactive	Inactive	Inactive	Inactive
Walking on treadmill	10 minutes	10 minutes	15 minutes	15 minutes	15 minutes	20 minutes	20 minutes	20 minutes
	40%	50%	50%	55%	55%	55%	60%	70%
	MHR	MHR	MHR	MHR	MHR	MHR	MHR	MHR

MHR: Maximum heart rate

**Table 2:** Strength exercises model

**Exercise kind**	**Week 1**	**Week 2**	**Week 3**	**Week 4**	**Week 5**	**Week 6**	**Week 7**	**Week 8**
Knee extension	Intensity 50% 1RM	Intensity 55% 1RM	Intensity 60% 1RM	Intensity 60% 1RM	Intensity 65% 1RM	Intensity 65% 1RM	Intensity 70% 1RM	Intensity 70% 1RM
	3 times’	3 times’	3 times’	3 times’	3 times’	3 times’	3 times’	3 times’
	10 repetitions each time	10 repetitions each time	10 repetitions each time	10 repetitions each time	10 repetitions each time	10 repetitions each time	10 repetitions each time	10 repetitions each time
Rest time	5 minutes	5 minutes	5 minutes	5 minutes	5 minutes	5 minutes	5 minutes	5 minutes
	Inactive	Inactive	Inactive	Inactive	Inactive	Inactive	Inactive	Inactive
Knee flexion	Intensity 50% 1RM’	Intensity 55% 1RM’	Intensity 60% 1RM’	Intensity 60% 1RM’	Intensity 65% 1RM’	Intensity 65% 1RM’	Intensity 70% 1RM’	Intensity 70% 1RM’
	3 times’	3 times’	3 times’	3 times’	3 times’	3 times’	3 times’	3 times’
	10 repetitions each time	10 repetitions each time	10 repetitions each time	10 repetitions each time	10 repetitions each time	10 repetitions each time	10 repetitions each time	10 repetitions each time

1RM: One repetition maximum

- Stage 3: one of the trainers demonstrated some simple stretching movements to ensure that all participants cooled down at the end of exercise sessions. Participants were further encouraged to take some fruit juice, date, biscuits, and milk.^35^

Ethical issues (including plagiarism, informed consent, research misconduct, data fabrication and/or falsification, double publication and/or submission, redundancy, etc.) have been completely observed by the authors. The Ethics Committee of Sport Science Research Institute of Iran approved the study protocol with Code No: S/93/398. For ethical reasons, at the end of the study the control group also received combinational exercises. All participants gave informed consent (both oral and written) in accordance with the declaration of Helsinki.

The normality of data was tested and confirmed by Kolmogorov-Smirnov test. Homogeneity of the four groups at baseline was confirmed using one-way ANOVA. Values from pre- (5 days before the intervention) and post-test (3 days after the intervention) were compared based on paired t-test. All data were analyzed using SPSS software (version 22, SPSS Inc., Chicago, IL, USA). An α-level of < 0.05 was considered significant.

## Results

**Figure 1:**
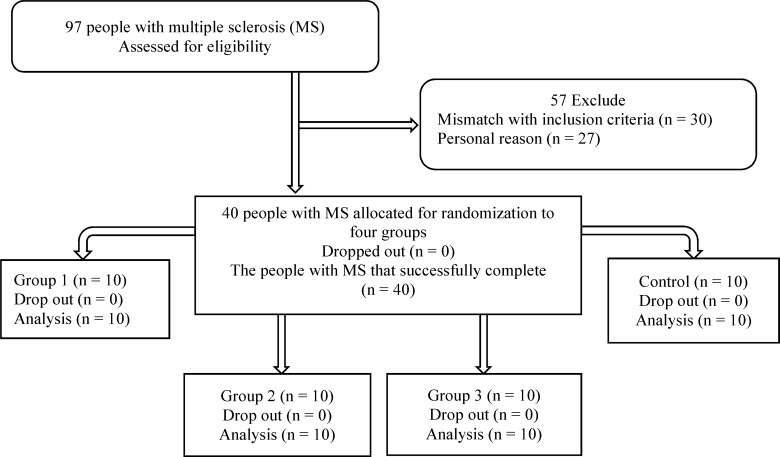
Consort flowchart

IMSS referred 97 RRMS patients to our rehabilitation center. According to the inclusion/exclusion criteria, 40 patients (24 female and 16 male) participated in the study; with mean disease duration of 2 years and BMI range of 18.5-25 kg/m^2^. All the patients successfully completed the procedure (Figure 1). There were 4 men and 6 women in each group. Table 3 gives descriptive statistics for age, height, weight, BMI and EDSS variables, separately for each group. 

***Test results***

6MWT‎: 6MWT score of the control group and the intervention group 1 did not change significantly comparing pre- and post-intervention values, whereas both intervention group 2 and 3 showed significant changes (P < 0.050) (Table 4). A comparison of post-test scores changes between groups declared a significantly higher score for groups 1 and 2 compared to the control group (P < 0.050).

10MWT: For 10MWT, all the three experimental groups showed a significant decrease in time taken to walk after the intervention (P < 0.050) (Table 4). The decreases in groups 1 to 3 were 2.4, 1.5 and 1.9 seconds, respectively. An average change in time taken to walk for group 1 was significantly different from control group (P = 0.030) (Table 5).

20MWT: In the ‎20MWT, time taken to walk significantly decreased after the intervention in experimental groups 1 (P = 0.045), 2 (P = 0.012) and 3 (P = 0.014) compared to the baseline values (day-0) (Table 4). In the control group, however, no significant change was observed (Table 4). An average change in time taken to walk for group 1 was significantly different from control group (P = 0.020) (Table 5).

**Table 3:** Mean of age, height, weight, body mass index (BMI), and expanded disability status scale (EDSS) for all groups

**Groups**	**Age (years)**	**Length (cm)**	**Weight (kg)**	**BMI (kg/m** ^2^ **)**	**EDSS (score)**
				
	**Mean ** **‎** **± SD**	**Mean ** **‎** **± SD**	**Mean ** **‎** **± SD**	**Mean ** **‎** **± SD**	**Mean ** **‎** **± SD**
Experimental 1 (n = 10)	35.80 ± 8.42	166.48 ± 6.99	68.13 ± 9.48	24.92 ± 2.76	1.33 ± 0.66
Experimental 2 (n = 10)	31.33 ± 8.21	164.97 ± 7.90	63.55 ± 13.65	23.99 ± 5.78	2.06 ± 0.86
Experimental 3 (n = 10)	33.91 ± 7.94	165.06 ± 8.56	66.92 ± 12.35	24.01 ± 3.35	1.95 ± 1.12
Control (n = 10)	33.63 ± 6.92	165.12 ± 7.59	63.00 ± 11.25	24.44 ± 4.78	1.81 ± 0.53

SD: Standard deviation; BMI: Body mass index; EDSS: Expanded disability status scale

BBS: BBS score significantly raised (about 6 points) after the intervention only for group 1 (P = 0.010) (Table 4). Group 1 score change was also significantly higher compared to the control group (P < 0.001) (Table 5).

Right knee extension and flexion strength (dominant leg): Right knee extension strength significantly increased in experimental groups 1 and 3 (P < 0.050). However, only group 3 showed a significant improvement of flexion strength (P = 0.012) (Table 4). A comparison of mean score change (post- and pre-score) indicated that flexion strength changes for intervention groups were significantly different from control group (P = 0.020, P = 0.040 and P = 0.010, respectively) (Table 5).

Left knee extension and flexion strength (non-dominant leg): Extension strength score significantly changed in all the intervention groups (P < 0.050). Average scores increased 8.4, 5.7 and 8.6 kg in groups 1 to 3, respectively. Moreover, flexion strength significantly changed in groups 1 and 3 (P = 0.015 and P = 0.001, respectively) (Table 4). A comparison of mean change in flexion and extension strength between groups showed that in groups 1 and 3, changes were significantly different from that of the control group (P = 0.010) (Table 5).

TUG test and FSS: A statistical analysis of both TUG test and FSS values did not indicate any significant change within groups.

In addition, based on post-hoc analysis of mean score change, none of the pair-wise comparisons of intervention groups were significantly different in 6MWT, 10MWT, 20MWT, right and left knee extension/flexion (P > 0.050).

**Table 4:** Descriptive statistics for four studied groups’ variables before and after test

**Variable**	**Group**	**n**	**Average before test**	**SD**	**Average after test**	**SD**	**Difference between average after test and average before test**	**Difference percentage**	**P**
10MW speed (s)	Control	10	15.217	18.94777	15.122	19.02946	−0.095	−0.624281255	0.758
	Group 1	10	9.828	4.89645	7.422	2.42591	−2.4056	−24.47750259	0.040*
	Group 2	10	8.109	2.08783	6.567	1.29852	−1.5413	−19.00774467	0.037*
	Group 3	10	9.874	5.56309	7.949	5.55153	−1.925	−19.49564513	0.014*
20MW speed (s)	Control	10	29.085	32.02146	28.985	32.13234	−0.1	−0.343819838	0.908
	Group 1	10	19.953	10.04469	14.876	5.00254	−5.0777	−25.4479209	0.045*
	Group 2	10	17.124	4.31811	13.306	2.03388	−3.8175	−22.29353298	0.012*
	Group 3	10	17.248	5.31164	13.919	3.99213	−3.329	−19.3007885	0.014*
Balance (score)	Control	10	45.000	10.04277	45.000	9.74500	0	0	0.214
	Group 1	10	43.111	4.96096	49.000	2.34521	5.8889	13.65982311	0.010*
	Group 2	10	49.375	3.06769	50.625	1.84681	1.25	2.53164557	0.080
	Group 3	10	45.400	8.93433	48.500	4.99444	3.1	6.828193833	0.060
Left knee extension strength (kg)	Control	10	10.667	5.04645	11.333	6.43946	0.6666	6.249355471	0.146
	Group 1	10	12.000	5.3619	20.444	6.12599	8.4444	70.37	0.004*
	Group 2	10	19.000	10.01428	24.750	10.93814	5.75	30.26315789	0.029*
	Group 3	10	14.580	7.16377	23.200	8.70249	8.62	59.12208505	0.001*
Right knee extension strength (kg)	Control	10	14.667	3.26599	16.667	7.44759	2	13.63633264	0.458
	Group 1	10	12.111	5.1099	19.000	6.61438	6.8889	56.88087787	0.002*
	Group 2	10	21.375	9.31876	25.000	10.91526	3.625	16.95906433	0.340
	Group 3	10	16.000	6.8313	24.300	8.53815	8.3	51.875	0.001*
Left knee flexion strength (kg)	Control	10	5.346	2.761	4.917	2.61566	−0.42897	−8.024625538	0.2390
	Group 1	10	7.422	3.50955	13.000	4.03113	5.5778	75.150225	0.015*
	Group 2	10	12.375	4.89716	15.500	5.47723	3.125	25.25252525	0.151
	Group 3	10	7.060	2.49275	12.600	2.79682	5.54	78.47025496	0.001*
Right knee flexion strength (kg)	Control	10	8.205	3.55624	7.750	2.80624	−0.4555	−5.551154713	0.100
	Group 1	10	7.722	3.64958	12.333	4.74342	4.6111	59.71225816	0.080
	Group 2	10	13.375	5.15302	17.250	5.94619	3.875	28.97196262	0.098
	Group 3	10	8.850	2.80921	12.900	3.38132	4.05	45.76271186	0.012*
6MWT	Control	10	361.500	238.86757	367.500	258.75692	6.0000	1.659751037	0.249
	Group 1	10	380.222	136.77790	461.444	139.61206	81.2222	21.36177674	0.057
	Group 2	10	422.500	106.39012	491.500	108.79338	69.0000	16.33136095	0.034*
	Group 3	10	363.000	159.48319	396.500	154.32739	33.5000	9.228650138	0.043*

SD: Standard deviation; 6MWT: 6 minute walking test; 10MW: 10 m walk

* Significant at α level less than 0.05

**Table 5:** The groups compare to control group

**The tests**	**Groups**	**Mean difference**	**SE**	**P**
Left knee flexion	Control group			
	Group 1	−5.57	2.09	0.010^*^
	Group 2	−3.12	2.14	0.150
	Group 3	−5.54	2.04	0.010^*^
Right knee flexion	Control group			
	Group 1	−4.61	1.89	0.020^*^
	Group 2	−3.87	1.94	0.040^*^
	Group 3	−4.05	1.85	0.010^*^
Left knee extension	Control group			
	Group 1	−7.77	2.73	0.010^*^
	Group 2	−5.08	2.80	0.080
	Group 3	−7.95	2.68	0.010^*^
Right knee extension	Control group			
	Group 1	−4.88	3.48	0.170
	Group 2	−1.62	3.56	0.650
	Group 3	−6.30	3.41	0.070
Balance	Control group			
	Group 1	−5.88	1.80	< 0.001^*^
	Group 2	−1.25	1.85	0.500
	Group 3	−3.10	1.75	0.090
6MWT	Control group			
	Group 1	−75.22	28.21	0.010^*^
	Group 2	−63.00	29.03	0.040^*^
	Group 3	−27.50	27.54	0.330
10MW test	Control group			
	Group 1	2.31	1.04	0.030^*^
	Group 2	1.45	1.07	0.190
	Group 3	1.83	1.01	0.080
20MW test	Control group			
	Group 1	4.98	2.05	0.020^*^
	Group 2	3.72	2.11	0.090
	Group 3	3.23	2.00	0.120

Significant at α level less than 0.05‎, SE: Standard error; 6MWT: 6 minute walking test; 10MW: 10 m walk

## Discussion

Our study was a randomized clinical trial to evaluate the effects of proposed combined exercises to improve muscle strength, balance, walking distance, and motor agility in patients with MS. We can divide the result into four sub-categories to discuss; the first part relates to tests that evaluated the strength of flexor and extensor muscles of the knees. The second part includes the tests that evaluated features of walking. The third and fourth parts are balance and fatigue scales.

Our findings showed a significant improvement of measures in intervention group 1, for which the dominant activity was aerobic exercise. Furthermore, they were in accordance with the studies of Dalgas et al.,^5^ Le Page et al.,^8^ Kjolhede et al.,^9^ Motl et al.,^36^ and Sangelaji et al.,^19^ However, Hansen et al.^37^ reported ineffectiveness of combined exercises, which may be due to application of different methods and measures as he used heart rate and blood examination. Significant strength improvements were observed in almost all knee flexor and extensor muscles in groups 1 and 3, but not group 2. These findings are in concordance with those of DeBolt and McCubbin,^38^ Kjolhede et al.,^9^ Le Page et al.^8^ and Medina-Perez et al.^39^ Although one would expect increase in muscle strength through resistance exercises, it was remarkable to note the increase in muscle strength with endurance exercises for group 1. A reason could be the fact that exercises such as stationary bike and treadmill walking may strengthen people with MS who lack regular exercise. This may also be a reason why significant improvement was detected for the non-dominant leg (left) and not the dominant leg (right). 

Walking features, namely duration and speed were tested by 10MW and 20MW tests as well as 6MWT. The results showed a pattern of effectiveness for aerobic exercises. For all three tests, group 1 showed a greater improvement compared to control group. In addition, a significant change was observed for group 2 in 6MWT. These results are in agreement with those of other studies including Cakt et al.,^40^ Rampello et al.,^16^ Geddes et al.,^41^ Motl et al.,^36^ Sangelaji et al.,^19^ and van den Berg et al.,^42^ which showed an improvement in walking endurance and speed after combined exercises. In addition, a systematic review by Citaker et al.^43^ showed a small significant change in mobility after exercise therapy. Hansen et al.,^37^ however, showed that combined exercises have no effects on endurance. The discrepancy between results could be explained by the differences in employed exercises and mobility measures. It seems that aerobic exercises such as treadmill can improve the gait style of the people with MS as well as their endurance and strength. With respect to our study design, no specific balance exercise was performed by the patients but the results showed a significantly greater post-intervention balance score for group 1 compared to both baseline and control group. The difference in the scores of two groups which reached nearly 6 points (P = 0.001) is noteworthy. This is in line with a study by Donoghue and Stokes^44^ that investigated the changes in Berg test corresponding to real changes in patients. 

In addition, our results matches, other studies like Paltamaa et al.,^21^ Kjolhede et al.,^9^ Sangelaji et al.,^19^ and Tarakci et al.^23^ In a study by Tarakci et al., the main reason for a significant difference between intervention and control groups was the odd point decrease of BBS in the control group in just 12 weeks and no increase in intervention group. It seems that treadmill as an aerobic exercise has some collateral effect such as balance improvement. Due to the nature of this kind of physical activity, some muscles that are effective in balance such as erector spinal muscles,^23^ may have strengthen and this phenomena may lead to improve balance in group 1 only and no the other groups. Studies by DeBolt and McCubbin^38^ and Rietberg et al.^7^ were in line with this concept. Some recent studies have focused on more specific muscles, such as Cakt et al.,^40^ Cattaneo et al.^20^ and Citaker et al.^43^ they all confirmed the effectiveness of resistance exercise on balance. Our results did not show any significant effect on fatigue. 

Fatigue is one of the most complicated symptoms in MS and the results of various studies on fatigue are contradicting. For instance, Sangelaji et al.,^19^ Cakt et al.,^40^ Schmidt and Wonneberger^45^ and Tarakci et al.^23^ reported a mild to moderate effect of aerobic, resistance and combined exercises on fatigue; however, van den Berg et al.,^42^ Rietberg et al.,^7^ Hansen et al.,^37^ and Geddes et al.^41^ did not find any significant effect of various type of exercise on fatigue. In addition, Surakka et al.^46^ reported a significant effect of aerobic activities on fatigue just in females. This discrepancy may be a result of varying interventions, insufficient intervention periods and examined population. Although TUG test score changes in groups 1 and 3 were significantly before and after the intervention, no significant change was detected in comparison with control group. Motl et al.,^36^ and Golzari et al.,^17^ however, showed that combined exercises have a significant effect on TUG test. This paradox could be due to the small sample size of intervention groups.

Main highlights of the present study are the choice of accessible exercises, use of a randomized controlled trial (RCT) study design and collaboration of various professionals (e.g., neurologist, physiotherapist, physical educator and epidemiologist).

Two major limitations were inability to control for diet attitude or sleep-wakefulness schedule of the participants. Furthermore, mood state of the participants was not controlled during the study course. In addition, because of space and time limitations, groups did their exercises in different sessions, so we could not match the groups based on the exact time of physical activity.

## Conclusion

Our study showed that a combined exercise schedule with a predominant aerobic component was more effective. The proposed model may help people with MS and can lead to improved balance skills, better walking abilities, and enhanced muscle strength. Furthermore, all modalities used in this model are simple, convenient and feasible. Hence, the proper combination of aerobic exercises with smaller portions of resistance exercises may be much more suitable for patients with MS. On the other hand, we showed a tangible improvement in test scores and scales after the intervention, specifically for groups 1 and 3; so we may speculate that rehabilitation and exercise therapy can help people with MS even in short-term. Finally, RCTs with large sample size and various exercise combinations are recommended to select the best rehabilitation regimen for people with MS.

## Notes:

**How to cite this article:** Sangelaji B, Kordi M, Banihashemi F, Nabavi SM, Khodadadeh S, Dastoorpoor M. A combined exercise model for improving muscle strength, balance, walking distance, and motor agility in multiple sclerosis patients: A randomized clinical trial. Iran J Neurol 2016; 15(3): 111-20.
